# Microstructure and Mechanical Behaviors of Titanium Matrix Composites Containing In Situ Whiskers Synthesized via Plasma Activated Sintering

**DOI:** 10.3390/ma11040544

**Published:** 2018-04-02

**Authors:** Yi Sun, Jian Zhang, Guoqiang Luo, Qiang Shen, Lianmeng Zhang

**Affiliations:** State Key Laboratory of Advanced Technology for Materials Synthesis and Processing, Wuhan University of Technology, Wuhan 430070, China; sunyiwhut@163.com (Y.S.); zhangjian178@whut.edu.cn (J.Z.); sqqf@263.net (Q.S.); lmzhang@whut.edu.cn (L.Z.)

**Keywords:** titanium matrix composite, plasma activated sintering, crystalline boron, amorphous boron, mechanical property

## Abstract

In this paper, titanium matrix composites with in situ TiB whiskers were synthesized by the plasma activated sintering technique; crystalline boron and amorphous boron were used as reactants for in situ reactions, respectively. The influence of the sintering process and the crystallography type of boron on the microstructure and mechanical properties of composites were studied and compared. The densities were evaluated using Archimedes’ principle. The microstructure and mechanical properties were characterized by SEM, XRD, EBSD, TEM, a universal testing machine, and a Vickers hardness tester. The prepared composite material showed a high density and excellent comprehensive performance under the PAS condition of 20 MPa at 1000 °C for 3 min. Amorphous boron had a higher reaction efficiency than crystalline boron, and it completely reacted with the titanium matrix to generate TiB whiskers, while there was still a certain amount of residual crystalline boron combining well with the titanium matrix at 1100 °C. The composite samples with a relative density of 98.33%, Vickers hardness of 389.75 HV, compression yield strength of up to 1190 MPa, and an ultimate compressive strength of up to 1710 MPa were obtained. Compared with the matrix material, the compressive strength of TC4 titanium alloy containing crystalline boron and amorphous boron was increased by 7.64% and 15.50%, respectively.

## 1. Introduction

Titanium-based metal matrix composite (TMC) has been widely used in the fields of aviation, aerospace, the chemical industry, and others because of its high specific strength, specific rigidity, and excellent mechanical properties at high temperatures [1,2,3]. Its reinforcement can be divided into continuous and discontinuous according to the state of the reinforcement (particles, whisker, or short fiber). Tomoyuki et al. have prepared alumina (Al_2_O_3_) titanium (Ti) composites to produce biocompatible materials with superior mechanical properties. The Al_2_O_3_–Ti composites were fabricated without any reaction phases, irrespective of raw materials [4]. The discontinuous nature of reinforced titanium matrix composites has attracted much attention for its isotropic, low cost, and simple preparation method.

The preparation process of TMCs can be divided into direct addition and in situ reaction by means of the addition of reinforcement [5,6,7]. Although the preparation process of direct addition is relatively quick and simple, the luster of titanium brings a series of issues, such as the degree of wetting [8,9,10], the reaction interface [11,12], a higher manufacturing cost than that of ordinary materials, and others [13,14]. In situ synthesis can generate one or several enhanced phases through an in situ reaction under certain conditions. This can be used to prepare particle-reinforced composites with a small particle size, a stable thermodynamic performance, no interface pollution, and a high bonding strength. It is a promising particle-reinforced composite material manufacturing process [15,16,17]. Research shows that TiB whiskers have as good physical properties and mechanical properties as in situ particles of TMCs. The interface between TiB and the Ti matrix introduced using the in situ, autogenous method is clean and has no interface reaction [18,19,20]. Chandravanshi et al. have studied the effect of boron on the microstructure and mechanical properties of thermomechanically processed near-alpha titanium alloy. The results indicated that the typical cleavage fracture mechanism at room temperature was the same as that at 550 °C [21]. Its good compatibility makes TiB an ideal reinforcement for titanium matrix composites.

TMCs are mainly prepared by powder metallurgy (PM), the casting solidification molding method, the spray molding method, and the laminated composite method [22,23,24]. For the base metal, a type and size of more than two kinds of second phase particle can be chosen according to the technical demands of the powder metallurgy composite method. However, the reaction between the matrix metal and strengthening particles is hard to achieve through the traditional hot pressing process [25]. Plasma activated sintering (PAS) is a technology developed in recent years for the synthesis of materials. Using the DC pulse voltage of the switch to generate an instantaneous, high-temperature plasma between the powder particles or the gap, it is possible to quickly eliminate the impurity and gas adsorbed on the surface of the powder particles, and promote the high speed diffusion and migration of the material. Ghasali et al. have compared the effects of spark plasma, microwave, and conventional sintering on the relative density and mechanical properties of Al-15 wt % TiC composite samples, and the SPS technique achieved the most remarkable effect [26,27]. The materials can be consolidated at lower temperatures, in a short period of time using the PAS process [28,29,30].

In this paper, the plasma activated sintering method was applied to the preparation of in situ titanium matrix composites by powder metallurgy. The influence of the PAS process and second phase particles used for the in situ reaction on the microstructure and mechanical properties of the prepared titanium matrix composites were investigated.

## 2. Experimental Procedure

**Figure 1:**
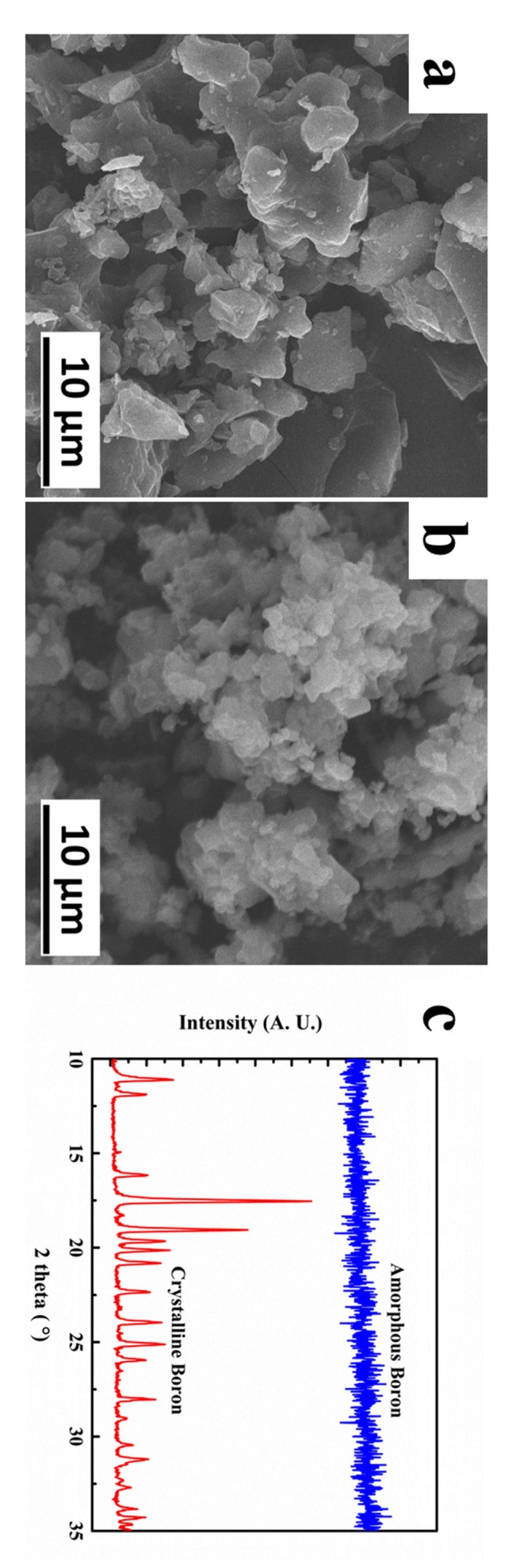
(**a**) SEM secondary electron image of crystalline boron (C-B) powder; (**b**) SEM secondary electron image of amorphous boron (A-B) powder; (**c**) XRD patterns of C-B and A-B powders.

In this work, spherical TC4 titanium alloy powders (6.1% Al, 4.3% V, 0.16% Fe, 0.01% C, remainder Ti; wt %) manufactured by the inert gas atomizing process were used as the raw material; the particle sizes were in the range of 15–45 μm; −400 mesh crystalline boron (C-B) and amorphous boron (A-B) powders were used as the second phase powder. The SEM images and XRD patterns of the two boron powders are shown in Figure 1. The design percentage of boron was 5 wt %. The TC4 titanium alloy powders were first mixed with 5 wt % boron powder in a shaker-mixer for 24 h. The milled powders were compacted into a graphite die with an inside diameter of 25 mm. PAS was carried out on the ED-PAS III system. Before sintering, the chamber was evacuated to a high vacuum. An uniaxial pressure of 20 MPa was employed during sintering, and the heating process consisted of two steps: a pulse electric current of 20 V and 100 A was loaded for 30 s to activate the surfaces of the particles, and then a direct current was passed through, the graphite die containing the powders was heated up at a rate of 100 °C/min, and held for 3 min at the design temperature [31,32,33,34].

The density of the sintered samples was measured by Archimedes’ principle. The phase identification was carried out by X-ray diffraction (XRD, Ultima III, Rigaku, Japan) with Cu Kα radiation. The microstructure of the polished surface and compressive fracture morphology of the samples were examined by field-emission scanning electron microscopy (FESEM, Quanta-250, FEI, Hillsboro, OR, USA). Vickers hardness was measured on the polished sections of the samples using Vickers indentation (Tukon 1202, Buehler, Binghamton, NY, USA) at a load of 500 g held for 15 s. The compressive strength (cylindrical specimens, of which the height was 10 mm and the diameter was 5 mm) was measured at an ambient temperature using a universal testing system (MT810, MTS Systems Corporation, Eden Prairie, MN, USA); the load speed during compressive test was 1 mm/min. Each sample was tested three times with nominally identical specimens to obtain an average value of the yield strength.

## 3. Result and Discussion

### 3.1. Relative Density

**Figure 2:**
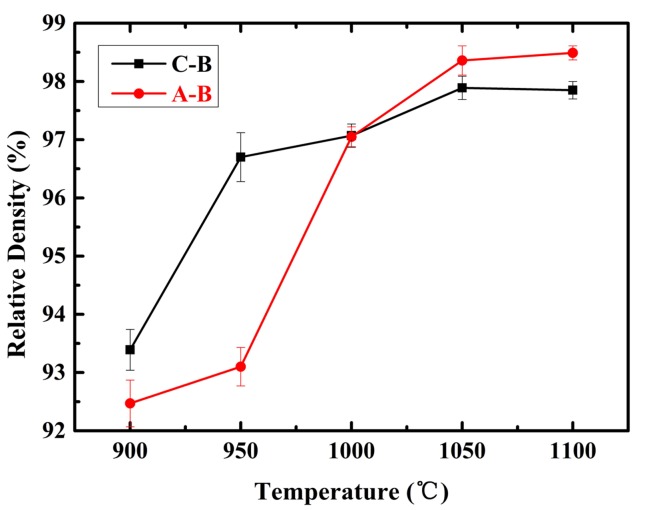
The relative densities of titanium-based metal matrix composites (TMCs) sintered at different temperatures.

Figure 2 shows the relative densities of TMCs sintered at different temperatures. The results indicate that the densities of the samples show an upward trend with the increase of the sintering temperature in the range of 900 to 1100 °C. The densities of the two kinds of composites are 93.40% and 92.46% at 900 °C, respectively; they then rise to 97.78% and 98.55% at 1100 °C. The relative densities of the samples with A-B are higher than the samples with C-B when the sintering temperature is higher than 1000 °C; this was due to the better chemical activity of A-B than C-B, thus allowing A-B to be more fully diffused and reacted with the matrix. The densities did not continue to rise obviously when the sintering temperature was higher than 1050 °C.

### 3.2. Microstructures

**Figure 3:**
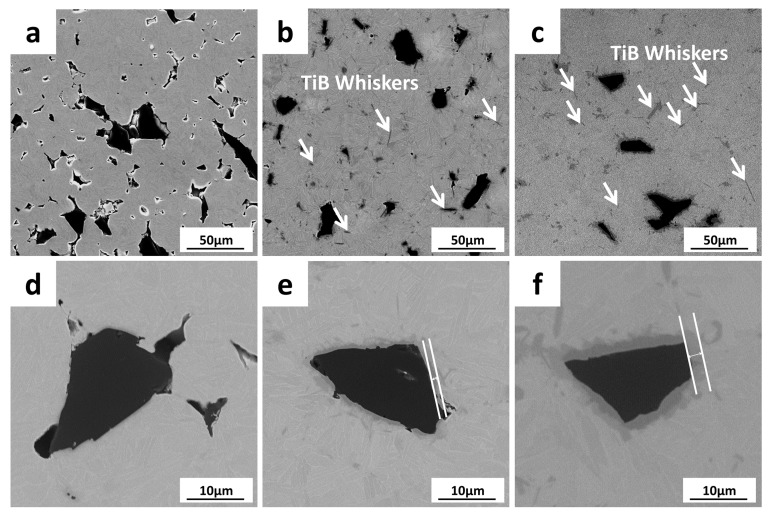
The microscopic morphology of TMCs with C-B addition sintered at different temperatures. (**a**) and (**d**) 900 °C; (**b**) and (**e**) 1000 °C; (**c**) and (**f**) 1100 °C.

Figure 3 shows the microscopic morphology of the samples with the addition of C-B at different sintering temperatures, and the combination of the particles with the matrix. The results show that at the sintering temperature of 900 °C, the boron particles were not tightly bonded to the matrix. When the sintering temperature rises to 1000 °C, the C-B particles were bonded closely with the matrix, and the reaction layer appeared. The thickness of the reaction layer was 1.26 μm, but pores could still be observed at the boundary. TiB whiskers were dispersed in the matrix; these were the complete reaction product of C-B and the Ti matrix. At the sintering temperature of 1100 °C, the intensity of TiB whiskers increased obviously. The reaction layer between the residual elemental boron particles and the matrix became thicker, measuring up to 3.35 μm. There were no obvious existing defects at the interface between the C-B particles and the matrix.

**Figure 4:**
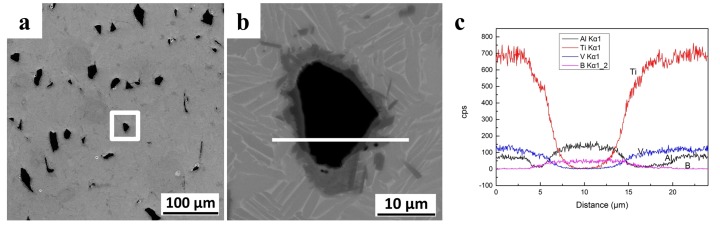
(**a**,**b**) The selected area of EDS analysis; (**c**) The EDS line of the micro-area at the second phase particle.

**Figure 5:**
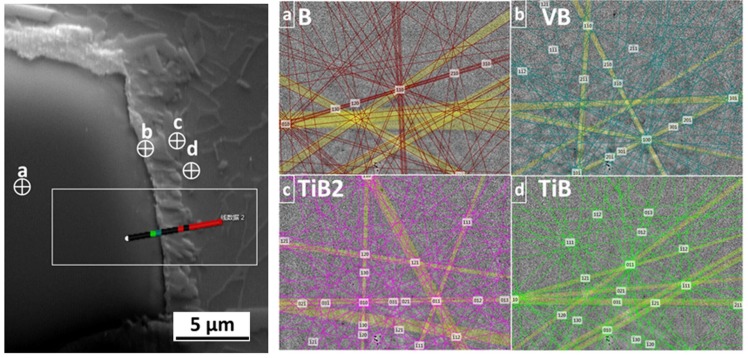
Micro-area EBSD phase analysis of the interface between C-B and the matrix. (**a**) C-B; (**b**) VB; (**c**) TiB_2_; (**d**) TiB.

EDS analysis was carried out on the micro-area; the distribution of elements is shown in Figure 4. The main elements in the diffusion layer were titanium, vanadium, and boron; the content of aluminum was low. Therefore, the diffusion layer was mainly formed by the diffusion and mutual reaction of these three elements. The micro-area EBSD phase analysis can be seen in Figure 5. The results show that with the diffusion between C-B and the matrix, the presence of B, Ti, TiB, and TiB_2_ can be observed at the interface. When the titanium element is in a supersaturated state, the reactant TiB_2_ will continue to react with the titanium to form TiB; the saturated state of the C-B elements near the side of the C-B particles does not provide the necessary conditions for any further reaction.

**Figure 6:**
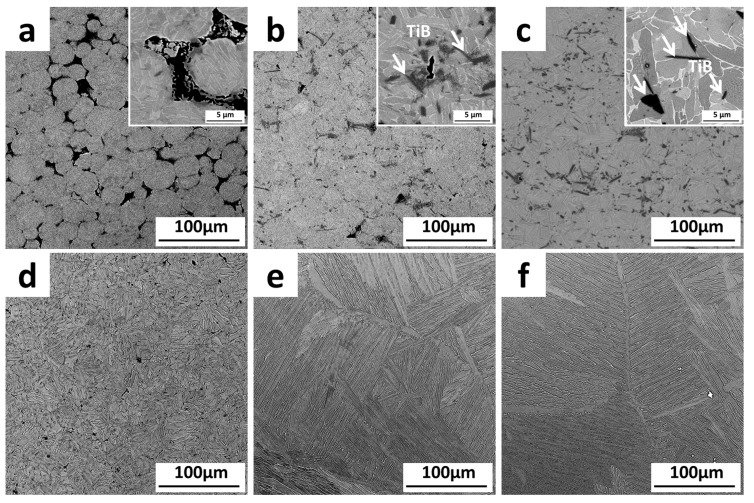
The microstructures of TMCs with A-B addition sintered at different temperatures: (**a**) 900 °C; (**b**) 1000 °C; (**c**) 1100 °C; and without addition: (**d**) 900 °C; (**e**) 1000 °C; (**f**) 1100 °C.

Figure 6 shows the microstructure of the composites with A-B addition at different sintering temperatures, and the microstructure of the matrix without the addition of the reinforcing phase at the corresponding sintering temperature. The results show that at 900 °C, the shape of the raw material TC4 powder was still legible; A-B particles distributed around the TC4 powder; few TiB whiskers could be observed in the matrix. However, the blank control group sample was fully saturated. When the sintering temperature was increased to 1000 °C, the bonding of the matrix powder was good, and most of the A-B species disappeared. However, the agglomeration of boron could still be observed in some areas, while reactant TiB whiskers were distributed in the matrix material. The grain shape of the material was a mixture of short plate and equiaxed, while the control group sample transformed into a whole lamellar tissue. The samples were nearly saturated with no obvious pores present at the sintering temperature of 1100 °C, and the boron was completely reacted with the matrix and distributed evenly in the matrix. The microstructure of the composites was still of an equiaxial and short-plate shaped mixed state. These phenomena were due to the fact that, in the control group samples, the powder was in contact with the same species that facilitates the formation of the sintered neck and the inter-diffusion of the elements. With the addition of the enhanced phase, the boron particles were uniformly wrapped around the matrix powder, which hindered the diffusion between the matrix powders, making it difficult to achieve sintering densification. Research showed that TiB whiskers could also pin the grain boundary effectively, preventing the grain from growing at high temperature. When its major axis is parallel to the grain boundary, TiB whiskers have the most remarkable pinning effect on the grain growth [35].

**Figure 7:**
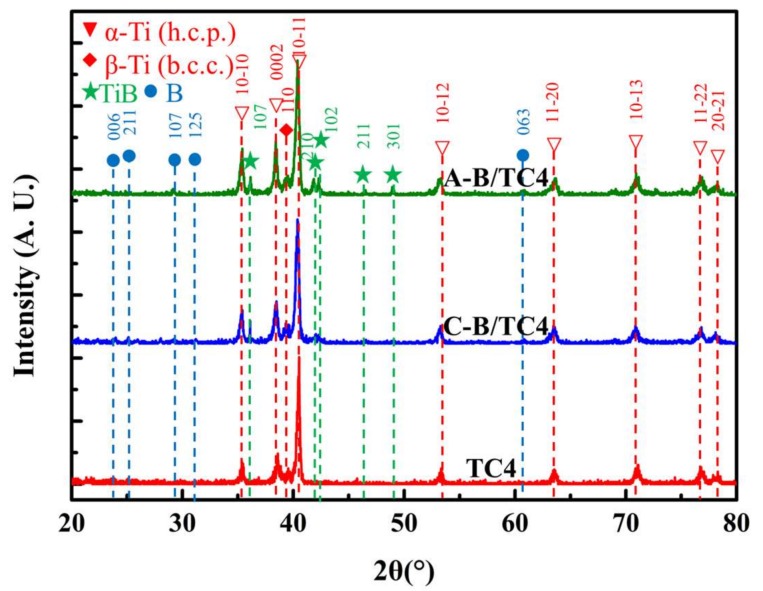
The XRD patterns of sintered samples.

The crystal structure of the specimens with different crystallographic boron addition were characterized by X-ray diffraction; all the samples were prepared at 1100 °C. The XRD patterns are displayed in Figure 7. The patterns indicate that the diffraction peaks of TiB could be observed in both the A-B–TC4 composite sample and the C-B–TC4 composite sample. The sample with A-B addition showed higher TiB diffraction peaks than the C-B–TC4 sample at the peak positions of the (107), (210), and (102) crystal faces. Several diffraction peaks of B can be found in the XRD pattern of the C-B–TC4 composite material, while there are no obvious peaks at the corresponding position of the A-B–TC4 composite sample. The complete reaction of A-B and the titanium matrix led to the higher content of TiB, while the typical B diffraction peaks in the C-B–TC4 composite sample are due to the amount of residual crystalline boron.

### 3.3. Mechanical Properties

**Figure 8:**
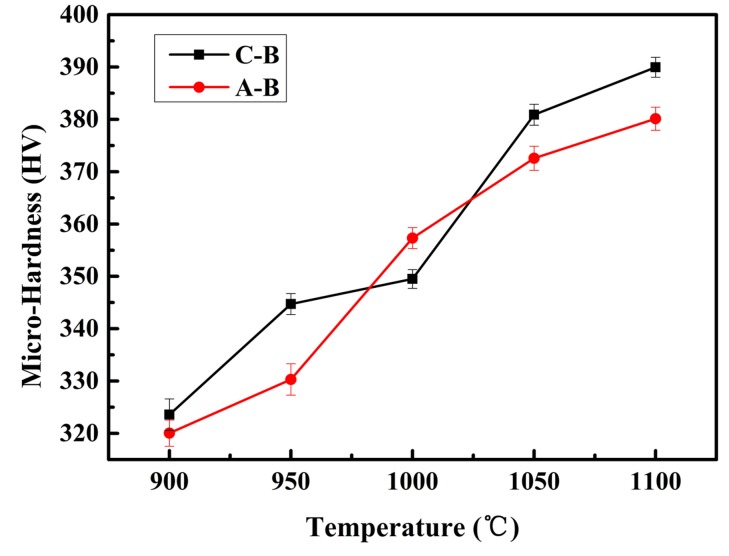
The Vickers hardness values of samples sintered at different temperature.

Figure 8 shows the Vickers hardness value of samples sintered at different temperature. The addition of A-B led to a more pronounced upward trend. A-B was more fully involved in the reaction under the same conditions, resulting in a higher volume fraction of TiB whiskers, and leading to an increase in grain boundaries. The high-density boundaries block the movement of dislocations because of the dislocation entanglement phenomenon; this improved the deformation resistance. Because the value of Vickers hardness was calculated by the area of indentation and the test pressure [36], the higher density of boundaries provided by the full reaction between A-B and the matrix contributed more to the hardness of the samples than that of the incomplete reaction of C-B.

**Figure 9:**
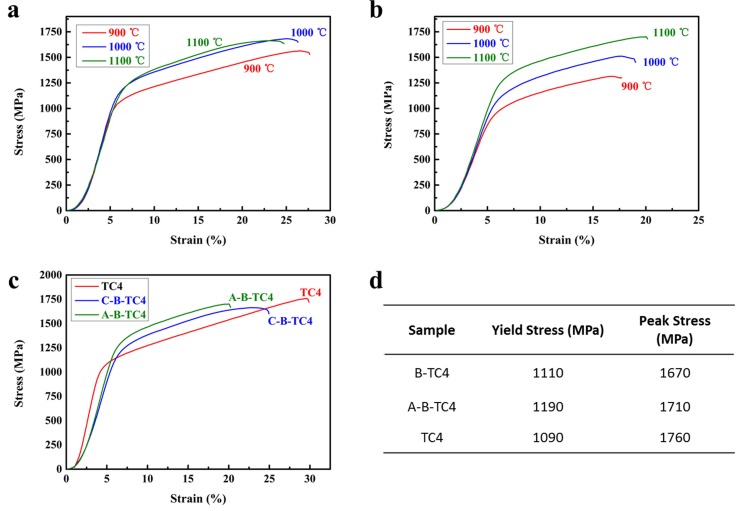
The compression strength curves of different groups of samples, (**a**) with C-B addition; (**b**) with A-B addition; (**c**) comparison between TMCs and the matrix material; (**d**) specific node value.

Figure 9a shows the compressive properties of the C-B addition samples prepared at different sintering temperatures. The compressive strength rose as the sintering temperature increased. This was because a high sintering temperature promotes the interface reaction between the element and the matrix. The same phenomenon occurred in Figure 9b, which shows the compressive strength curves of the A-B addition samples. Figure 9c is a comparison of the compressive strength curves of the composite material prepared at 1100 °C with the boron-free matrix material. The results show that the compressive strength of the sample with the enhanced phase is significantly higher than that of the matrix material, and the yield strength was increased by 8.56%. Huang et al. have studied the transformation of TiB whiskers during plastic deformation. Their result showed that a significant recrystallization occurred in the primary α phase of titanium matrix composite materials. When true strains reached 1.81%, the dislocation density decreased obviously. An equiaxed microstructure was obtained after the complete recrystallization of the alloy, and a good effect on the comprehensive performance of the material was reported [35].

**Figure 10:**
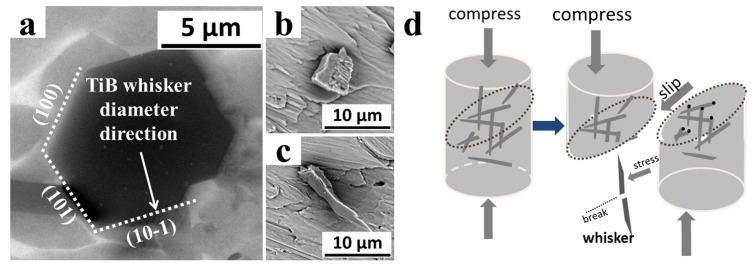
(**a**) The hexagonal cross section of the in situ TiB whisker; (**b**,**c**) TiB whiskers on the sliding surface; (**d**) The fracture form of the TiB whiskers during the slip of the compression.

With the increase of the sintering temperature, A-B reacts with the matrix to form TiB whiskers. These in situ whiskers can enhance the performance of the sample in the process of compressive fracture when compared with TC4 titanium materials with similar compositions. Sun et al. have prepared a TC4 titanium block with the same microstructure of axial and short, tabular grains as the matrix, and obtained a compression stress of 1020 MPa, 7.8%, lower than the composite material in this paper [37]. Figure 10 shows the fracture form of the TiB whiskers during the slip of the compression. The cross section of the in situ TiB whiskers was of a hexagonal shape; (100), (101) and (10-1) crystal planes were clarified in Figure 10a. The growth in the longitudinal direction of the whisker is aligned in the (010) direction according to the earlier studies [21]. These whiskers aligned in different directions provide shear stress and axial tension stress as shown in Figure 10b,c, while the strain rate of the sample also increased due to the hindering and entangling of the dislocation brought about by the second phase particles.

**Figure 11:**
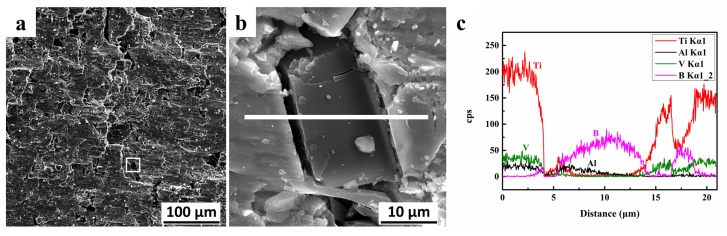
(**a**,**b**) The compression fracture surface of the sample with C-B addition; (**c**) EDS line of the selected area.

Figure 11 shows the compression fracture surface of the sample with C-B addition, and the state of the second phase particles during the fracture process. The EDS line indicates that the interface of the second phase has a small aluminum content, and the boron and vanadium elements are enriched. The reaction product of this region is mainly VB compound, which is a brittle ceramic phase with low intensity. A crack appeared in this region during the process of compression deformation. This crack occurred only in the vicinity of the grain, and did not expand to the matrix, playing a role in energy absorption and contributing to the plastic deformation of the material.

## 4. Conclusions

In this paper, titanium matrix composites with in situ TiB whiskers were synthesized by plasma activated sintering; the effect of C-B and A-B addition as the reactant were compared and investigated. 

Titanium matrix composites containing in situ whiskers with a high relative density were obtained under the PAS condition of 20 MPa and 1000 °C for 3 min. A-B had a higher reaction efficiency than C-B; for this reason, it could completely react with the titanium matrix to generate TiB whiskers, while C-B still had a certain amount of residual particles at 1100 °C. The in situ whiskers obtained a length in the range of 2~10 μm, and the microstructure of the matrix was of an equiaxial and short, plate-shaped mixed state.

The composite samples obtained high comprehensive mechanical properties with a Vickers hardness of 389.75 HV, a compression yield strength of up to 1190 MPa, and an ultimate compressive strength of up to 1710 MPa. The in situ whiskers helped the titanium matrix composites maintain an equiaxed and short plate-like uniform microstructure distribution, and enhance the compression strength through the dislocation entanglement phenomenon, which contributed to the good compressive mechanical properties.
